# Repeated cytoreductive surgery (CRS) with hyperthermic intraperitoneal chemotherapy (HIPEC) in patients with recurrent peritoneal carcinomatosis

**DOI:** 10.1186/s12957-016-0804-x

**Published:** 2016-02-24

**Authors:** Nikolaos Vassos, Thomas Förtsch, Archil Aladashvili, Werner Hohenberger, Roland S. Croner

**Affiliations:** Department of Surgery, University Hospital Erlangen, Krankenhausstrasse 12, 91054 Erlangen, Germany; National Cancer Center of Georgia, Tbilisi, Georgia

**Keywords:** Cytoreductive surgery, CRS, Repeated, Iterative procedure, HIPEC, Peritoneal surface malignancy, Recurrent peritoneal carcinomatosis

## Abstract

**Background:**

Cytoreductive surgery (CRS) with hyperthermic intraperitoneal chemotherapy (HIPEC) has become the treatment of choice for resectable peritoneal carcinomatosis (PC) and improved the survival of these patients. The situation changes if PC recurs and repeated CRS with HIPEC is considered. The patient selection and outcome of the repeated approach has not been well described. We analyzed our cohort and share the experiences.

**Methods:**

Ninety-three CRS/HIPEC procedures, performed in 85 patients during the period 2001–2013, were examined in a retrospective analysis. Type of primary, ECOG status, peritoneal cancer index (PCI), completeness of cytoreduction (CC), duration of hospitalization, postoperative morbidity, mortality, and disease-free/overall survival were reviewed.

**Results:**

Six patients (7 %) underwent a second CRS/HIPEC (median interval between the two procedures: 26 months, range 8–61) including two patients with mesotheliomas, one patient with ovarian adenocarcinoma, one patient with leiomyosarcoma of uterus, one patient with colon adenocarcinoma, and one patient with appendiceal adenocarcinoma. The last two patients underwent a third CRS/HIPEC, 25 and 36 months, after the second procedure. The median PCI was 14 (range, 4–26) during the first and 20 (range, 7–39) during the second CRS/HIPEC of these patients. Completeness of cytoreduction score of 0 (CC-0) was achieved in all first procedures and in 67 % of second procedures (CC-0; *n* = 4 and CC-1; *n* = 2). A CC-0 score was possible in both of the third procedures. The mean operating time was 444 min (range, 198–642) and 427 min (range, 239–617) during the first and the second procedure. Median intensive care unit (ICU) was 2 days, and hospital stay after second CRS/HIPEC was 17 days (range, 7–50). The 30-day morbidity after repeated CRS/HIPEC was 33 % (16 % for grade III–IV complications), and there was no 30-day mortality neither after the second nor after the third CRS/HIPEC. Median disease-free interval between first CRS/HIPEC and peritoneal recurrence was 17 months (range, 8–30). Median disease-free survival of 18 months (range, 4–33) was achieved after the second CRS/HIPEC. After a median follow-up of 74 months (range, 39–151), all patients are alive with disease (*n* = 5) or disease free (*n* = 1) under chemotherapy.

**Conclusions:**

In experienced centers, repeated CRS/HIPEC can be performed with safety. Patient selection and correct timing is of particular importance in achieving control of the disease. Repeated CRS/HIPEC should be considered as treatment option for selected patients with recurrent PC.

## Background

Peritoneal carcinomatosis (PC) had traditionally been treated as part of palliative phase and resulted in a poor prognosis with fatal disease progression. However, cytoreductive surgery (CRS) followed by hyperthermic intraperitoneal chemotherapy (HIPEC) has nowadays been established as a treatment option for this situation and remains important in the repertoire of managing these patients [1, 2]. The principle underlying this therapeutic modality is the complete resection of all visible macroscopic peritoneal metastatic disease, followed by the administration of HIPEC, which provides higher local concentration of the chemotherapeutic agents and additional cytotoxic effect of the hyperthermia, resulting in treatment of any residual, microscopic peritoneal disease [3, 4]. This procedure is indicated when peritoneal disease is confirmed intra-abdominally and often used in peritoneal disease from appendiceal neoplasms, mesotheliomas, and ovarian or colorectal cancer, or even gastric cancer, sarcomas, and primary peritoneal carcinoma [5–10].

Due to the progression of surgical technologies and techniques, the morbidity and mortality rates of such treatment approaches in high-volume centers have decreased accordingly with a corresponding increase in the overall survival of selected patients with peritoneal surface malignancies [6–12]. Multiple studies showed an effectiveness of CRS/HIPEC reporting that 5-year survival rates for pseudomyxoma [6, 13–16], PC of colorectal origin [7, 17, 18], PC of ovarian origin [8, 19, 20], and peritoneal mesothelioma [10, 21, 22] ranges from 50 to 96 %, 22 to 51 %, 17 to 55 %, and 29 to 63 %, respectively.

However, approximately 80 % of patients with PC of colorectal origin, 24–44 % of patients with pseudomyxoma and 40 % of patients with mesothelioma recur alone in the peritoneal cavity after CRS/HIPEC [13, 14, 23–28]. In these cases, the option of a potentially curative repeated CRS/HIPEC becomes a possible consideration. However, limited data on outcomes and survival has been published regarding recurrence of PC treated with repeated CRS/HIPEC, and indications still remain unclear.

The objective of the study is to perform a retrospective analysis of patients at our institution who underwent repeated CRS/HIPEC as treatment of recurrent peritoneal carcinomatosis after primary CRS/HIPEC. We aim to evaluate the utility and feasibility of repeated CRS/HIPEC by recording the outcome, morbidity, and mortality of the procedure proposing that patients who undergo multiple CRS/HIPEC have improved long-term outcomes with similar morbidity and mortality to the first CRS/HIPEC.

## Methods

### Clinicopathological data and parameters and outcome measures

A retrospective study of all patients who suffered recurrent disease after primary CRS/HIPEC and underwent repeated CRS/HIPEC procedure at our institution was conducted. Between 2001 and 2013, 93 CRS/HIPEC procedures were performed in 85 patients. Four patients underwent two CRS/HIPEC and two patients underwent three CRS/HIPEC procedures. Repeated CRS/HIPEC was recommended for patients with histological evidence or suspicion of recurrent peritoneal disease, based on clinical, laboratory (elevated tumor markers: CEA, CA125), and radiological examinations. These patients were discussed at the multidisciplinary tumor board, and the final decision was made by consensus, taking into consideration the patients related variables as well as parameter which could represent contraindications. The patients’ selection criteria for repeated CRS/HIPEC are shown in Table 1.

**Table 1:** Patients’ selection criteria for repeated CRS/HIPEC

Inclusion criteria	Exclusion criteria
Histologic or cytologic diagnosis of PC	Extraperitoneal or liver metastases
Complete recovery from prior systemic chemotherapy or radiation treatments	Poor performance status (ECOG 2–3)
Tolerance of initial HIPEC without major morbidity	Multiple small bowel obstruction
Disease considered to be resectable based upon imaging	Biliary obstruction
Good performance status (ECOG 0–1)	Short bowel syndrome
Prior R0 or R1 resection during the first CRS	Severe malnutrition
Maintain of nutritional reserves (albumin >3 g/dl)	Short disease-free interval
Favorable tumor biology	Class III appearance of the small bowel
Interval between two procedures of 6–12 months	

Patient data as age, race, gender, and ECOG-graded functional status were reviewed. Furthermore, operative variables as date of initial and repeat CRS/HIPEC, type of primary malignancy, chemotherapeutic agent, hospital and intensive care unit (ICU) length of stay, morbidity, mortality, and disease-free and overall survival were recorded for each patient.

Τhe peritoneal cancer index (PCI), as previously described by Sugarbaker, was used to determine the extent of peritoneal disease. Completeness of cytoreduction score (CC-score) was also recorded and was graded from a score of 0–3, measuring the amount of disease left behind after CRS [2, 29]. Surgical complications were defined and classified as minor (grade 0/I/II) and major (grade III/IV) according to Clavien-Dindo’s classification of surgical complications [30]. Treatment-related mortality was classified as death within 30 days of surgery during hospitalization.

The follow-up period commenced at the date of initial surgery with the censor date of December 2015. Two disease-free survival (DFS) were computed: time from initial CRS/HIPEC to first recurrence and time from second-time CRS/HIPEC to second recurrence or end-time of analysis (December 2015). Overall survival (OS) was calculated from the date of initial CRS/HIPEC and from the date of repeated CRS/HIPEC to the date of death from any case or to the end-time of analysis.

### CRS/HIPEC procedure

The treatment consisted of two elements: aggressive surgical cytoreduction and HIPEC. All patients underwent exploratory laparotomy, and CRS was performed as described by Sugarbaker; it consists of six peritonectomy procedures and resection of all macroscopic visible peritoneal disease aiming to attain a complete cytoreduction (CC-0) [29]. The objective of repeated CRS/HIPEC remained similar. Following the CRS procedure, HIPEC was performed using closed technique for 60 min via the inflow and outflow catheters placed during the operation. Chemotherapeutic agent was infused in a body surface area-based (BSA-based) dose (mitomycin C 20 mg/m^2^ or cisplatin 50 mg/m^2^) at 42 °C using the hyperthermia pump. Mitomycin C was the drug of choice for PC from primary colorectal and appendiceal carcinoma, and cisplatin was the drug used when the primary was ovarian carcinoma, sarcoma, peritoneal mesothelioma, or primary peritoneal carcinoma. The same agent was administrated in the first and the repeated CRS/HIPEC procedure as well. Patients were monitored in the intensive care unit during the first 24 h of the postoperative period or until stabilization and were subsequently transferred to the surgical oncology floor.

## Results

### Patients

From 2001 to 2013, six patients underwent repeated CRS/HIPEC procedures for isolated peritoneal tumor recurrence and were included in our study. These six patients represent a highly selected subset (7 %) from 85 patients who underwent totally 93 CRS/HIPEC procedures. Seventy-nine patients did not undergo any iterative CRS/HIPEC procedure because either they appeared no recurrent peritoneal disease or they were not considered to iterative CRS/HIPEC procedure. The distribution of primaries in these 79 patients was as follows: colorectal carcinoma (*n* = 29), pseudomyxoma (*n* = 11), appendiceal (*n* = 9) and ovarian (*n* = 9) carcinoma, adenocarcinoma of stomach (*n* = 9), mesothelioma (*n* = 5), primary peritoneal serous papillary carcinoma (*n* = 3), small intestine cancer (*n* = 2), ewing sarcoma (*n* = 1), and leiomyosarcoma of uterus (*n* = 1).

Of 85 patients, a total of 25 patients (29.5 %) had a recurrent peritoneal disease after the first CRS/HIPEC procedure in a median interval time of 20 months (range, 3–58). Of these patients, 19 patients were not considered to repeated/iterative CRS/HIPEC. The reasons for exclusion were extraperitoneal or liver metastases (*n* = 10), disease considered to be unresectable upon imaging (*n* = 7) with poor performance status (ECOG 2 or 3), CC-score of 2 during the laparotomy (*n* = 2), and short bowel syndrome (*n* = 1).

Our high selected subset consisted of six patients. The mean age of patients was 48 years (range, 16–64). There were five females and one male patient. The primary tumors were as follows: malignant peritoneal mesothelioma (*n* = 2), ovarian carcinoma (*n* = 1), leiomyosarcoma of uterus (*n* = 1), colon carcinoma (*n* = 1), and appendiceal carcinoma (*n* = 1). The median interval time from diagnosis to first CRS/HIPEC was 15 months (range, 1–62), from the first to second CRS/HIPEC was 26 months (8–61), and from the second to third CRS/HIPEC was 30 months (25–36, *n =* 2). Three patients did not receive preoperative systemic chemotherapy before the first HIPEC, and a systemic chemotherapy was not performed before the repeat HIPEC in three patients.

### HIPEC and perioperative parameters

The median PCI score during the first CRS/HIPEC was 14 (range, 4–26), and complete cytoreduction was achieved for all six patients with a final cytoreduction score of 0 (CC-0). During the second CRS/HIPEC, the median PCI score was 21 (range, 11–39), and CC-0 cytoreduction was achieved in the majority of patients (*n* = 4, 67 %); the other two patients were able to achieve a CC-1 score. All patients undergoing a third CRS/HIPEC (*n* = 2) achieved a CC-0 having a median PCI score of 22 (range, 19–25).

Length of surgery tended to be shorter in those who underwent repeated CRS/HIPEC 427 min (range, 239–617) vs. 444 min (range, 198–642) in initial CRS/HIPEC, although this was not statistically significant. In the third CRS/HIPEC, the length of surgery was 711 min (range, 503–919). The required ICU time after first and second CRS/HIPEC was similar too (1.8 vs 1.6 days) whereas median hospital stay on the surgical floor was 13 days (range, 10–17) for first, 17 days (range, 7–50) for second, and 26 days (range, 9–43) for third CRS/HIPEC procedures, respectively, without any significant difference in ICU and hospital stay time between initial and repeat HIPEC.

In our study, two of our six patients developed postoperative complications after the second CRS/HIPEC showing a postoperative morbidity rate of 33 % whereas the morbidity of our complete patient cohort (*n* = 85) after one CRS/HIPEC was 21 %. The complications after the repeated CRS/HIPEC were as follows: (a) renal failure (grade I), resolved after conservative treatment, and blood transfusion (grade II) in the patient 1 and (b) enterocutaneous fistula with intra-abdominal hemorrhage (grade III) in the patient 3, requiring relaparotomy; the patient was discharged in good condition after a prolonged hospital stay. Hence, the morbidity of major complications requiring invasive or surgical management (grade III) was 16 % (*n* = 1) in our case series study. There was no postoperative mortality.

The details of all six patients are summarized in Table 2.

**Table 2:** Summary table of treatment and outcomes of our cohort

Patient	1	2	3	4	5	6
Primary malignancy	Colorectal	Uterine sarcoma	Mesothelioma	Appendiceal	Ovarian	Mesothelioma
Gender	F	F	M	F	F	F
Age	52	64	60	48	48	16
First CRS/HIPEC						
PCI score	4	21	26	16	10	11
CC score	0	0	0	0	0	0
Disease-free interval (DFI), (months)	8	11	24	25	10	30
Interval between first and second CRS/HIPEC, (months)	8	16	24	61	15	31
Second CRS/HIPEC						
PCI score	7	27	22	39	20	11
CC score	0	0	0	1	1	0
Disease-free interval (DFI) (months)	25	4	5	30	10	33
Overall survival since initial CRS (months)	71	39	69	151	48	64
Overall survival since second CRS (months)	63	23	43	102	33	33
Total number of CRS/HIPEC	3	2	2	3	2	2
Current status	Alive, stable disease under chemo therapy	Alive, disease regression under chemotherapy	Alive, progressive disease under chemotherapy	Alive, stable disease under chemotherapy	Alive, progressive disease under chemotherapy	Alive, no evidence of disease under chemotherapy

### Survival

Five patients (83 %) and four patients (67 %) received adjuvant chemotherapy after initial and repeat HIPEC, respectively. A median DFS (disease-free survival) of 18 months (range, 8–30) was achieved after initial CRS/HIPEC till disease recurrence was detected. All patients in this study cohort had a disease-free duration of at least 6 months after the first CRS/HIPEC, and the administrated chemotherapeutic agent was not replaced by another agent. A median DFS of 18 months (range, 4–33) was achieved after repeated procedure till second recurrent disease. Of the six patients of our study, five patients recurred after the second CRS/HIPEC with a median disease-free interval (DFI) after the second CRS/HIPEC of 14.8 months (range, 4–30), two of them (uterine leiomyosarcoma, peritoneal mesothelioma) without a direct avenue for further treatment. Two of the patients who recurred after the second CRS/HIPEC (adenocarcinoma of colon, appendiceal carcinoma) underwent a third CRS/HIPEC after 25 and 30 months, respectively. The first patient received further chemotherapy after the third CRS/HIPEC, was free of tumor for 16 months, and at the last follow-up had a stable disease under chemotherapy. The second one received no chemotherapy after the third CRS/HIPEC and was free of tumor for 24 months till recurrent peritoneal metastases were diagnosed and treated by chemotherapy (at last follow-up stable disease). Regarding the subgroup of patients (*n* = 79) who did not undergo repeated CRS/HIPEC, the disease (local recurrence and metastases)-free survival was 21.8 months (range, 3–104).

Median follow-up time (overall survival), which captures to death or last follow-up, was also recorded for patients undergoing repeat HIPEC. The median follow-up time from diagnosis of primary tumor for patients undergoing repeat HIPEC was 89 months (range, 64–152) and median follow-up since the first CRS/HIPEC was 74 months (range, 39–151), whereas the median follow-up time since the second CRS/HIPEC was 50 months (range, 33–102). To date, no patient was lost to follow-up and all patients are still alive undergoing chemotherapy treatment. Two patients had a stable disease, two patients progressive disease, and one patient with disease regression; the last one remained free of disease under chemotherapy. The overall survival of the patients without treatment via repeated CRS/HIPEC (*n* = 79) was 26.8 months (range, 3–108). To date, 45 patients were alive (57 %), 27 died of disease (35.5 %), five died during the hospital stay (postoperative mortality: 6 %), and one was lost to follow-up (1.5 %).

## Discussion

Peritoneal carcinomatosis (PC) cannot any longer be considered as an unresectable, terminal metastatic disease since there is growing evidence that patients affected by PC benefit from CRS/HIPEC procedure [6, 12, 21, 27]. But a subset of PC patients present with recurrent disease confined to the peritoneal cavity after the first procedure which could be explained by the advanced stage of the disease at the time of diagnosis [24–27]. Managing these patients is challenging with no established protocols and no clear evidence of treatment modalities for recurrent PC after primary CRS/HIPEC. High-volume centers approach recurrent disease with heterogeneous strategies, additive chemotherapy, radiotherapy, iterative CRS (debulking), or repeated/iterative CRS/HIPEC (iCRS/HIPEC) [23, 26], and there has been a reasonable discussion regarding the tolerability of repeated CRS/HIPEC due to the extent of the operation and the clinical and physiologic impact on the patient. There have been a few studies noting improved long-term overall survival for patients who underwent only second-look surgery without HIPEC after the first CRS/HIPEC procedure, compared to survival rate for patients who did not receive second CRS procedure [31, 32].

Recently, repeated CRS/HIPEC has been also evaluated in the setting of recurrent peritoneal disease in some studies with variable results [24, 26, 27, 32–42]; but improved long-term survival was generally suggested (Table 3). In our study of 85 patients, six patients (7 %) were selected to undergo iCRS/HIPEC. This rate is compatible with the rates of other studies from specialized centers (4–8 %) [24, 27, 33, 34]. However, Sugarbaker et al. [43] and Chua et al. [35] reported the largest cohorts of patients who were able to undergo a second CRS/HIPEC (26 % 124/472 and 16 % 79/466, respectively). This treatment option is performed and justified in highly selected patients with an intention to achieve further disease control and to prolong the survival of patients [32]. The patients we considered to be the best candidates for a successful repeat cytoreduction are those who filled out the criteria presented in Table 1, such as completeness of initial cytoreduction, interval between initial HIPEC and recurrence, and good functional status. In our study, the CC-0 of all initial cytoreductions, the median interval of 26 months between the two procedures, and the ECOG 0 status of patients mirror the consideration of these criteria. These various strict criteria and patient factors must be evaluated by the multidisciplinary tumor board.

**Table 3:** Comparable illustration of studies’ results concerned clinical and survival parameters in repeated CRS/HIPEC

Study	Number	type of primary	PCI	CC-0/1 (%)	Median time between first and second HIPEC (months)	Length of stay (days)	30-day morbidity (%)	30-day III/IV morbidity (%)	30-day mortality (%)	Median follow-up after repeated HIPEC (months)	Median overall survival after repeated HIPEC (months)	1J (%) after repeat HIPEC	3J (%) after repeat HIPEC	5J (%) after repeat HIPEC
Lubrano (2006) [41]	5	Various^a^	n.a.	n.a.	n.a.	n.a.	30	n.a.	0	n.a.	n.a.	60	40	20
Brouquet (2009) [24]	20	Various^b^	6	n.a.	17^j^	15	60	30	5	63.2	n.a.	96	82	72.5
Saxena (2010)[42]	40	Various^c^	n.a.	66	n.a.	32	80	35	2.5	n.a.	n.a.	n.a.	n.a.	n.a.
Golse (2012) [33]	30	Various^d^	8	90	22	15	73.3	40.5	3.3	18	140^l^	n.a.	n.a.	n.a.
Votano poulos (2012) [27]	62	Various^e^	9	43.5	17^j^	7.5	48.4	33.3	3.2	60.8	32.3	78.7	48.6	31.6
Chua (2013) [35]	79	Various^f^	16	92.4	n.a.	29	n.a.	41	0	24	48	90	60	34
Sardi (2013) [37]	26	Appendiceal carcinoma	23	65	23	11	n.a.	42	0	28	46.5	90.9	54.3	33.9
Wong (2014)[36]	8	mesothelioma	13	100	15.6	8	50	25	0	56.7^k^	80^k^	100^k^	88^k^	64^k^
Iheme landu (2015)[38]	44	mesothelioma	14	34.1	12.5	14	29.6	2.3	0	31^k^	54^k^	n.a.	61^k^	46^k^
Vaira (2014) [40]	16	Various^g^	n.a.	n.a.	13^j^	n.a.	43.7	18.7	n.a.	n.a.	n.a.	n.a.	n.a.	n.a.
Wong (2015) [39]	7	Various^h^	12	100	20^j^	12	28.5	14	0	13	20.7	85	43	29
Vassos (2016)(current study)	6	Various^i^	6	100	25.8	17	33	16	0	50	50	100		

*n.a.* not available

^a^n.a.

^b^Pseudomyxoma, colorectal (CRC), mesothelioma, carcinoid

^c^Pseudomyxoma, CRC

^d^Pseudomyxoma, CRC, mesothelioma, ovarian cancer, gastric cancer, cholangiocarcinoma, leiomyosarcoma, primary peritoneal carcinoma

^e^Appendiceal carcinoma, CRC, mesothelioma, ovarian cancer, gastric cancer, GIST, gallbladder carcinoma, small bowel carcinoma, leiomyosarcoma, urachal carcinoma

^f^Pseudomyxoma, CRC, pseudomyxoma, appendiceal carcinoma, small bowel carcinoma, ovarian cancer, hepatocellular carcinoma

^g^Pseudomyxoma, CRC, mesothelioma, ovarian cancer

^h^Appendiceal carcinoma, CRC, mesothelioma, ovarian cancer

^i^Appendiceal carcinoma, CRC, mesothelioma, ovarian cancer, uterine sarcoma

^j^Median time till diagnosis of recurrent PC

^k^Time period estimating from first CRS/HIPEC

^l^Time period estimating from diagnosis

Repeated CRS/HIPEC is a technically feasible surgical option but the risks of iterative procedures must be carefully evaluated in addition to the potential survival benefit. The morbidity rate of 33 % and the mortality rate of 0 % in our series are comparable with that experienced in our patients undergoing an initial CRS/HIPEC (21 and 1 %, respectively). Interestingly, the grade III/IV morbidity is only 16 % (8 % after initial CRS/HIPEC) which is one of the lowest reported in the literature (Table 3), whereas no difference was observed in complication rates among patients treated with different HIPEC regimens [24, 27, 33, 35].

In our study, two patients underwent three CRS/HIPEC and both of them were free of tumor for 16 and 24 months, respectively, having a stable disease at the last follow-up. Three or more CRS/HIPEC procedures were already reported by other authors [32, 37]. Sardi et al. reported four patients who underwent a third CRS/HIPEC and a 1-year survival rate of 75 % [37], similar to the 5-year OS of 70 % and a 10-year survival of 53 % for patients undergoing three or more CRS/HIPEC reported by Mohamed [44].

Our study also shows that systemic chemotherapy is valuable as an additional single-treatment modality. We suggest that systemic chemotherapy should always be considered in addition to a secondary cytoreduction since preoperative chemotherapy could help the selection of good candidates, improve the resectability rate of PC, and decrease the recurrence rate after CRS/HIPEC. However, there are currently no data to support this hypothesis, and, in the present series, the sample size was too small and the disease biology so variable to draw any conclusion. At any case, a median overall survival of 89 months (range, 64–152) from diagnosis was achieved and all of our patients were alive during the last follow-up remaining under control of disease through systemic chemotherapy. Interestingly, the youngest patient of our collective remained free of disease. This overall survival of 100 % in our case series study mirrors the consideration of strict selection criteria for repeated CRS/HIPEC, the role of the additional systemic chemotherapy, and the importance of early diagnosis of recurrence.

We must indeed highlight the importance of early diagnosis of recurrence. Chua et al. [45] recently suggested that careful follow-up for early detection of recurrence was an important requirement for management of colorectal peritoneal metastases via iterative CRS/HIPEC. We agree with this suggestion and believe that patients should be surveyed with the appropriated imaging at regular intervals of 3–6 months within the initial 5 years when most disease recurrence for those with PC would occur. This is to ensure that recurrences confined to the peritoneal cavity are diagnosed when they are still resectable and fairly low volume. In our series, most cases of recurrence was diagnosed with CT scan or/and serum tumor markers, in the absence of clinical symptoms.

Multiple studies have shown that survival after initial CRS/HIPEC was adversely influenced by different factors such as increased PCI, incomplete CC, high ECOG, poor nutrition, and histopathologic type of primary tumor [7, 18, 24, 32, 44–49]. However, there are no absolute independent exclusion factors for iCRS/HIPEC procedures. When evaluating the benefits of repeated CRS/HIPEC, completion of cytoreduction indicated by CC score, distribution, and volume of PC as defined by PCI and interval between the two procedures or even postoperative complication are important factors in predicting and prognosticating outcomes [12, 26, 28, 32, 38, 43]. Esquivel et al. [32] demonstrated a negative impact on survival if the PCI score of iCRS/HIPEC is increased supporting the use of iterative procedures in patients with limited PC [26, 45, 48]. However, if the disease could be removed completely, we often performed iCRS/HIPEC despite high-volume disease. CC scores from the initial CRS/HIPEC can also help identify patients for a repeat procedure. Our policy is to perform iCRS/HIPEC exclusively in patients with completely resected disease (CC-0) in the initial CRS [26]. Furthermore, CC-0/1 scores from repeated CRS can have a profound effect on outcome. If complete cytoreduction is deemed impossible on the exploration of abdomen in the iCRS, an aggressive intervention with HIPEC should be abandoned and an alternative approach pursued [31, 32]. Our study showed that a complete cytoreduction was feasible in both procedures. However, the ability to achieve a complete resection is dependent not only on tumor histopathology and tumor extension but also on operator expertise and skill.

We recognize that this study has several limitations since it has a small patient population and it is retrospective in nature. There is also a selection bias since the patients offered for repeat procedures were highly selected. Other confounding factors relate to the different chemotherapeutic regimens and the role of the systemic therapy on the behavior of isolated peritoneal disease. We do not claim that our results can be applied to every patient with peritoneal surface disease. However, we do believe that this treatment is feasible and safe for high-selected patients.

## Conclusions

In conclusion, our study proposes that repeated CRS/HIPEC have meaningfully good long-term outcomes with similar morbidity and mortality to that of initial CRS/HIPEC. Selecting not only the correct patient but also the correct timing to perform iterative procedures in high-volume tertiary care centers with expertise in the treatment of peritoneal surface disease is of paramount importance in achieving prolonged survival. Repeated CRS/HIPEC could be considered as treatment option for highly selected patients with recurrent PC.
